# Compliance with research ethics in epidemiological studies targeted to conflict-affected areas in Western Ethiopia: validity of informed consent (VIC) by information comprehension and voluntariness (ICV)

**DOI:** 10.1186/s12910-024-01003-5

**Published:** 2024-01-18

**Authors:** Gemechu Tiruneh, Mekdes Yilma, Bizuneh Wakuma, Eba Abdisa, Lami Bayisa, Michelle Nichols, Anja Bedeker, Nicki Tiffin

**Affiliations:** 1 Department of Public Health, Institute of Health Sciences, Wollega University, Nekemte, Ethiopia; 2 School of Nursing and Midwifery, Institute of Health Sciences, Wollega University, Nekemte, Ethiopia; 3 South African Medical Research Council Bioinformatics Unit, South African National Bioinformatics Institute, University of the Western Cape, Bellville, South Africa; 4 College of Nursing, Medical University of South Carolina, Charleston, SC USA

**Keywords:** Ethics, Conflict settings, Informed consent

## Abstract

**Background:**

The conduct of research is critical to advancing human health. However, there are issues of ethical concern specific to the design and conduct of research in conflict settings. Conflict-affected countries often lack strong platform to support technical guidance and monitoring of research ethics, which may lead to the use of divergent ethical standards some of which are poorly elaborated and loosely enforced. Despite the growing concern about ethical issues in research, there is a dearth of information about ethical compliance in conflict areas. Valid and ethically informed decision-making is a premier pact with research participants in settling possible ethical issues before commencing the research, which is ensured by gaining informed consent from prospective participants of the research.

**Aims:**

This research aimed to explore compliance with research ethics and consent validity in community-based epidemiological research conducted previously.

**Methods:**

Research participants were recruited in the western part of Ethiopia in three districts subjected to conflicts. A community-based cross-sectional study design was utilized, and 338 residents were enrolled as study participants. All participants had previously been enrolled as research participants in epidemiological studies. Data was collected using a questionnaire that was pilot-tested before the commencement of the main data collection. The questionnaire focused on participants’ experiences of the informed consent process followed when they were recruited for an epidemiological study and covered themes such as essential information provided, level of comprehension, and voluntarism of consent.

**Results:**

Over half of the study participants, 176 (52%), were not provided with essential information before consenting. And 135 (40%) of them did not comprehend the information provided to them. One hundred and ninety (56%) participants freely and voluntarily agreed to partake in one of these epidemiological studies, with over a quarter (97; 28.7%) of them reporting they were subjected to undue influence. Written consent was obtained from only 32 (9.4%) of the participants.

**Supplementary Information:**

The online version contains supplementary material available at 10.1186/s12910-024-01003-5.

## Background

Research has been a core activity in the development of mankind and scientific advancement. Research involving human subjects is required to observe universal principles of ethical consideration [1, 2]. However, as research becomes more complex and specialized, the ethical considerations that come with it become increasingly important. Compliance with ethical principles of research is therefore a universal act to be practiced in research involving human participants in any setting, including conflict-afflicted areas [2–5].

Although ethical review is a fundamental process for ensuring safe and ethical conduct of research, real-time assessment of adherence to ethical requirements remains challenging in field research in low- and middle-income countries (LMICs), especially within conflict-stranded contexts [5, 6]. Evaluating the quality of informed consent is deemed necessary to keep up with the required ethical standards in LMICs [7].

Conflicts are still common in many low-income and resource-constrained areas, posing multifaceted health problems in the lives of the inhabitants [8, 9]. The presence of armed conflict has largely limited medical research due to insecurity, limited infrastructure, and a lack of competent human power [10]. Therefore, this has led to a shortage of research evidence revealing challenges facing the health needs of populations in conflict areas [5].

When research is conducted in conflict settings, the design and ethical rules are subject to compromise due to the lack of strong platforms committed to monitoring research ethics. Lack of training on research ethics, the presence of violence limiting access to populations over time, and difficulty of onsite supervision and monitoring are some of the contributing factors leading to poor and ethical compromise in research addressing conflict settings [3, 10].

Despite the barriers hindering full-scale research implementations and the ethical challenges involved, there is a clear need to conduct targeted research to improve specific health interventions and shed light on the plight of people in conflict settings [3, 10]. However, there are some ethically important issues related to the design and conduct of research, particularly in conflict situations.

Community-based research in conflict-affected areas has been difficult to provide valid data because of the practical, methodological, and ethical challenges of research in conflict-affected areas [10, 11]. Despite growing concerns about ethical issues in research involving human subjects, there is a shortage of evidence on the ethical compliance of research in areas associated with conflict. There have been clear challenges in addressing the maintenance of research ethics in fields of limited security. A robust ethical framework must be put in place that not only protects participants but also keeps their opportunities open [10].

Informed consent is a willing and full decision to participate in research taken by a competent individual who has received adequate information about the research and is vital for ensuring respect for the individual’s autonomy [2, 12].

Informed consent is the acceptance of participation in research by a competent person who has received the necessary information needed to make an informed decision, fully understood that information, and is able to make decisions without coercion or undue influence. Obtaining informed consent from study participants before their enrollment in any research is a universally agreed standard and acts as a foundational basis for the ethical conduct of health care research [2, 12, 13]. Though there are other ways of authorization and seeking permissions, like community consultations and institutional permissions, individual consenting should not be overlooked and replaced with individual consenting unless waived by ethics committee in special circumstances [2].

The effectiveness of informed consent is mainly measured by three elements of the consent process: the nature and depth of the information provided to the participants, their comprehension of the information provided, and their willingness [12, 14].

Unlike the current study, previous studies [15] mainly focused on the comprehension element of consent, and most were facility-centered clinical trials. This study aims to verify compliance with research ethics and the validity of the consent process in terms of information provided, comprehension assessment, and voluntariness in community-based health research, targeting conflict areas in western Ethiopia.

## Methods

### Study contexts

This study took place in three districts in the west of Ethiopia, where previous epidemiological studies assessing the prevalence of malnutrition had been conducted. The three districts involved in those previous epidemiological studies of malnutrition and the current consent assessment study are *Leka Dulecha, Jima Arjo*, and *Wayu Tuka*. The parental study was intended to determine the magnitude of under-nutrition and factors associated in the three districts. The study subjects were under five and school age children. Anthropometric measurements were taken from all children and biological specimen from the school age children. During the interview, the parents participated as respondents and gave their consent on behalf of the children. The districts had been stranded by sustained and intermittent insurgencies that involved armed conflicts. The western part of Ethiopia has been flagged as insecure on national security maps [16], and the majority of the districts have been cut off from essential health coverage services during conflict times. Despite the evident security problems, some research projects are approved to be conducted by local universities and research institutes as part of routine research projects or research requirements for the award of master’s and doctoral degrees.

### The study design

This was a cross-sectional study and the study was conducted from December 2022 to March 2023. The study was community-centered, and the study participants were accessed by house-to-house visits.

### Study population and recruitment

The study sample was sourced from studies that occurred before the commencement of the current study. The previous studies determined the epidemiology of undernutrition among school-age children and children under the age of five. Parents were respondents and provided consent on behalf of the children during the epidemiological studies of undernutrition. For this consent assessment study, participants were recruited from parents who responded during the parental study. All participants had to be 18 years of age or older, which is the legal age for consent in Ethiopia. A total of 338 competent adults who fulfilled the preset inclusion criteria were included in the consent assessment survey. These respondents were recruited among the total of 1951 source population that took part in the previous parental study in the specified districts. The participants were selected conveniently based on availability and willingness of the respondents during a second ethics survey.

### Data collection tools and techniques

Data was collected using a survey guide tool developed for this study. Requirements in international ethical guidelines [2, 17] were considered while developing the tool. The study team drafted the first version of the tool. Only items applicable to the former studies were considered in developing the tool [Supplementary file 1]. The majority of the items had binomial response options. After iterative revisions, the final version of the tool was pilot tested on 30 individuals who had previously participated in research at public health facilities in Nekemte City. A reliability test was done on the pilot data. The Cronbach’s Alpha was + 0.78 for the twenty-two items of the tool. Minor amendments were made after piloting, and the tool was finally loaded into Kobo-Collect Version 2022.2.3, an Android-based data collection application run on smartphones. The use of the Kobo-Collect Android data collection application minimizes the probability of erroneous data collection compared to paper-based data collection techniques. The tool was pilot tested and redeployed for final data collection. Data collectors were trained on the tool, data collection process, ethics, and consent-taking procedures ahead of data collection.

### Data analysis and management

The collected data was submitted to a Kobo Toolbox account and imported into SPSS V.25 to be analyzed. Frequency statistics were run to identify outliers and the percentage of missing data per case and per item. The missing observations were Missing Completely at Random (MCAR) as verified by Little’s Missing Completely at Random test. The maximum number of missing observations was 1.5%, and all were imputed. Some of the variables were transformed and recoded into binomial variables. A descriptive analysis of frequency and percentages was used to present a summary of the information in the variables with a corresponding 95% confidence interval. Clopper-Pearson method was used to calculate the binomial confidence intervals. The responses in the information provision section were presented as either “informed” or “not informed” for each of the variables. In the comprehension assessment section, participants were subjected to picking the correct responses on generic domains of consent. The right responses, according to the information provided in the consent of the previous studies, were regarded as correct and indicated by “good comprehension, while incorrect responses were relabeled as “poor comprehension.” The bar graphs were plotted for data visualization.

## Results

### Socio-demographic profile of the study participants

The survey included 338 study participants, with a mean age of 30.5 years (SD = 5.5). The survey was conducted in three districts found in the western part of Ethiopia, in the eastern Wollega Zone of Oromia Regional State. Almost half (47.3%) of the study participants were from Jima Arjo District. Over half (55%) of respondents were male. The vast majority of respondents were married at the time of the survey (90%). 42% of participants had attended primary school. Almost a quarter (21.6%) of participants reported having no formal education. Farming was the most frequently reported occupation (54.7%), followed by merchanting (18.6%). [Table 1].

**Table 1:** Socio-demographic profile of the study participants

Variable	Categories	Frequency	Percentages
Districts of data collection	Jima Arjo	160	47.3
	Leka Dulecha	50	14.8
	Wayu Tuka	128	37.9
Sex of the respondents	Female	152	45.0
	Male	186	55.0
Marital status	Married	304	90
	Single	34	10
Educational attainment	No formal education attended	73	21.6
	Primary school attended (grades 1–8)	143	42.3
	Secondary School attended (grades 9–12)	79	23.4
	College and Above	43	12.7
Occupation	Day laborer	24	7.1
	Farmer	185	54.7
	Government Employee	45	13.3
	Housewife	21	6.2
	Merchant	63	18.6
Religion	Orthodox	154	45.6
	Protestant	184	54.4

### Type of information provided to the prospective participants

Participants were asked if they had been provided with particular information regarding the previous research they had participated in. Eight essential information points were administered, all of which were categorized as either “informed” or “not informed. Less than two-thirds (65%) of participants reported having been informed of the purpose of the research they participated in [95% CI 60–70%, p = 0.001]. Similarly, over half of respondents (55%), reported they did know or were informed of the eligibility criteria for their participation [95% CI 49.7–60.3%, p = 0.001]. Nearly half (56%) of respondents indicated they were clearly informed of the procedure to be carried out during the research process [95% CI 50.7–61.3%, p = 0.001]. Most respondents reported being informed that they could withdraw from the study (53%) [47.8–58.7%], while a similar number (52%) [46.6–57.6%] said they were not provided with information about confidentiality issues. 57% of the participants reported they had no information on the benefits associated with participation [95% CI, 51.3–62.2%, p = 0.012]. Additionally, 69% of the participants had no information about the risks of participation [95% CI, 63.4–73.6%, p = 0.012]. Additionally, 60% of them did not know or were not informed about their right to know their health status [95% CI, 54.7–65.2%, p < 0.001]. [Table 2]

**Table 2:** Type of information provided to the research participants

Type of information	Response category	Frequency(n)	Percentage [95% CI]	P-Value
Information regarding research purpose	Informed	220	65% [60 − 70%]	0.001
	Not Informed	118	35%	
Information on selection criteria	Informed	186	55% [49.7 − 60.3%]	0.001
	Not Informed	152	45%	
Information on procedure	Informed	189	56% [50.7 − 61.3%]	0.001
	Not informed	149	44%	
Information to withdrawal	Informed	180	53% [47.8–58.7%]	0.001
	Not informed	158	47%	
Information on Confidentiality	Not informed	176	52% [46.6 -57.6%]	0.001
	Informed	162	48%	
Benefit of Participation	Not informed	192	57% [51.3 − 62.2%]	0.001
	Informed	146	43%	
Risk of Participation	Not informed	233	69% [63.4 -73.6%]	0.001
	Informed	105	31%	
The right to be informed of the findings after research completion	Not informed	203	60% [54.7 − 65.2%]	0.001
	Informed	135	40%	

### The comprehension status of the research participants

The research participants were asked questions assessing their comprehension of domains of consent. Five domains of consent were provided, with ample response options for each. Correct answers, as per the previous consent administered, were denoted as good comprehension and incorrect responses as poor comprehension.

**Fig. 1:**
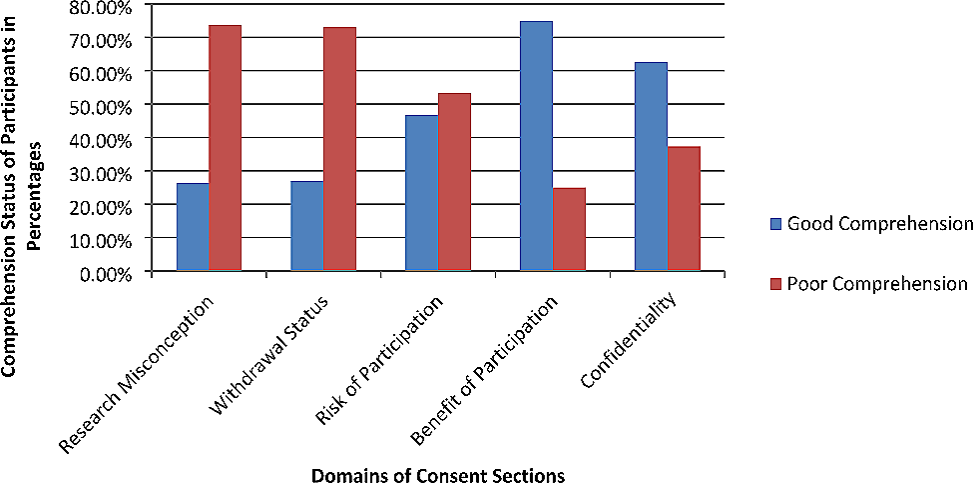
Bar graph depiction showing Comprehension status of the study participants

The five consent assessment domains in research misconception, withdrawal status, risk of participation, benefit of participation and confidentiality [Fig. 1].

In this study just over a quarter (26.3%) of the participants accurately understood the event (the malnutrition assessment survey) was intended for research data collection (26.3% [21.6–31%). Nearly the same number of participants (27%) correctly identified the possibility of withdrawal and autonomous participation (27% [22.5–32.5%]). Nearly half (46.7%) had good comprehension regarding all the risks associated with research (46.7% [41–52%]). Exactly three-fourths of the participants correctly identified the correct responses, demonstrating the benefit of enrolling in the parental study, which indicated good comprehension [95% CI 70–79.6%, *p* = 0.001]. Finally, 62.7% of the participants demonstrated good comprehension towards the confidentiality of the information provided: 62.7% [57.5–67.8%] *p* < 0.001. [Table 3]

**Table 3:** Comprehension status of the study participants

Domains of Consent	Response items	Frequency	comprehension Status	Percentage with [95% CI]	P-Value
Research misconception	Data collection for research	89 [26.3%]	Good Comprehension	26.3%[21.6-31%]	0.001
	Part of health care service	181[53.6%]	Poor comprehension	73.7%	
	Health related campaign	68[20.1%]			
Possibility of Withdrawal	Free to withdraw	92[27.2%]	Good Comprehension	27%[22.5-32.5%]	0.011
	Withdraw if granted permission	185[54.7%]	Poor comprehension	73%	
	No option of withdrawal	61[18%]			
Risks of Participation	The procedure was non-invasive and painless	158[46.7%]	Good Comprehension	46.7%[41-52%]	0.012
	The procedure was painful	39[11.5%]	Poor comprehension	53.3%	
	Did not understand risks	141[41.7%]			
Benefits of Participation	No direct benefit of partaking	62[18.3%]	Good Comprehension	75%[70-79.6%]	0.001
	Beneficial to the community	192[56.8%]			
	Not clearly understood	84[22%]	Poor comprehension	25%	
Confidentiality	The data handled securely	136[40.2%]	Good Comprehension	62.7%[57.5-67.8%]	0.001
	Un-named data is used	76[22.5%]			
	Not clearly understood	126[37.3%]	Poor comprehension	37.3%	

### Voluntarism status of the study participants

Another aim of the research was to explore the voluntarism status of the research participants. Although the majority of the participants, 190 (56%) indicated their participation was voluntary, 28.7% of the participants reported they felt pressured to consent to the research, and 15.1% felt subjected to undue influence for their participation. The vast majority of the participants (90.6%) provided oral consent, and only 9.4% of them signed to participate [87.5–93.7%]. Among study the participants in the previous study, biological specimen was received from 160 of the study participants, which were school age children. Three-fourths (75%) of the children’s parents’ were aware of and consented to the procedure on behalf of their children [67.6–81.5%], and the remaining part claimed they were not informed the children had to provide biological specimen hence, did not provide a fully informed consent to the biological specimen received from the children [Table 4].

**Table 4:** Voluntarism status of previous research participants

Type of information	Response category	Frequency	Percentage with 95% CI	P-Value
Status of Voluntarism	Voluntary Participation	190	56.2% [51-61.5%]	0.001
	Felt pressurized ^*^	97	28.7%	
	Felt Subjected to influence^$^	51	15.1%	
Consenting Mechanism	Provided oral consent	306	90.6%[87.5-93.7%]	0.001
	Provided written consent	32	9.4%	
Consent for Biological Specimen (*n* = 160)	Informed Consent	120	75% [67.6-81.5%]	0.001
	Uninformed Consent	40	25%	

* [ The participants reported slight form of pressure to partake in the research by the community health care workers, community representatives’ and health professionals participating in the research.]

$ [ The participants reported of being influenced in expect of medical benefit for their enrolled children].

## Discussions

To our knowledge, this is the first study to focus on ethical compliance with the aim of verifying consent validity in community-based epidemiological surveys in conflict-affected settings. The study focused on key elements of consent such as information provision, comprehension assessment, and voluntariness. Eight items were used to assess the type of information provided to the participants.

More than half of participants in this consent assessment study reported they had heard about withdrawal, inclusion criteria, the purpose, and the procedure of the study. Beyond that, half of them claimed no information about benefit, risk, confidentiality, or feedback about research. More than two-thirds of our study participants have known the purpose of the research, and this is related to a study from Uganda where three-fourths of participants reported they were told about the purpose of the research [18]. Although our study assessed discrete information as opposed to general adequacy, almost half of the study participants missed at least one of those items. This result looks lower compared to other studies that reported a high proportion of overall adequacy of information provision during consent [19, 20]. This variation is due to the context of the current study being community-based in a conflict area, compared to facility-based in the other studies.

Despite the availability of enlisted essential information in the ethics guidelines [2, 17] that is to be provided during consent, there is still a lack of consensus on the type and amount of information to be provided to declare adequacy [21, 22]. Therefore, in many studies, this has resulted in non-uniformity among items considered for the assessment of information. In some studies, the adequacy of information was generally assessed according to the participant’s declaration rather than an objective assessment [19, 20].

Observing the effectiveness of consent processes should be a compulsory effort for researchers by ensuring the comprehensibility of the information provided to enhance voluntary participation when taking consent on a study-by-study basis [23]. In this study, a discrete assessment was conducted on the comprehension status of the participants regarding research misconceptions, confidentiality, withdrawal status, and the risks and benefits of the study. Three-fourths of the participants did not realize they were participating in research. This finding is comparable to a study from Mali, which reported 74% non-differentiation of the research from routine care services [24]. Compared to the current study, significantly surpassing findings were reported from studies conducted in Nigeria and South Africa, which revealed that 85% and 92% of the study participants differentiated between research and routine care, respectively [25, 26]. Researchers are advised to raise the understanding of the participants before taking their consent. Though, it may be challenging to associate the poor comprehension with the conflict, there is a concern that lack of familiarity with research, lack of equivalent translation of research in local language, and limited access to health care can jeopardize the ability of study participants to fully differentiate research and routine care in developing countries and affect fully informed and voluntary consent [27–30]. On top of the prevailing conflict, all these hold true in the context of the current study area.

In this study, only a quarter of the participants demonstrated good comprehension of withdrawal. Related results were reported by a study from West Africa, which revealed that 21% of the participants said they could withdraw at any stage of the research [22]. A higher rate of understanding about withdrawal criteria was reported by other studies from East and South Africa, which were 64% and 87%, respectively [18, 26]. It is surprising to note that our finding is below the lowest value of the pooled estimate [33.3–78.6%] of withdrawal status comprehension in LMIC [15]. Quitting after enrollment was considered disrespectful to the researcher and staying to the end was regarded as compulsory by participants in another study [24]. The nature of the study being a trial and the better research infrastructure in the cited studies may also contribute to the observed variation among the studies.

Despite the noninvasive nature of the previous epidemiological research, which was not liable to palpable risk, more than half of the participants in our study revealed poor comprehension of risk. Some studies across West Africa have also reported a low rate of understanding about expected risks and drug side effects among the majority of the participants [24, 27]. However, the finding of our study is low compared to a study conducted in South Africa, which reported that 79% of participants were aware of the risks related to participation in the research [31].

Three-fourths of the participants correctly recalled that the study was useful to the community’s children of a comparable age group, even though they did not gain direct benefit from participation. The figure is encouraging among the other studied parameters. As any health-related campaign is deemed advantageous in resource-deprived areas, there is a high tendency to understand the benefits and remain cooperative in participation [24].

This study also assessed the participants’ understanding of their confidentiality status. The majority (62.7%) of the participants had good comprehension status. In another study reported, 85.8% of the participants did not know how their records would be kept and handled [27].

More than half of the participants in this study reported voluntary participation, and 30% of them felt pressured; the remaining felt influenced to participate. The majority of the data collectors and community mobilizers during the epidemiological study were service providers and community health workers, which could derail the willingness of the participants. Some of the participants may have been influenced to participate in the hope of receiving medical benefits for their participating children. Other studies also found that the majority of the participants were influenced to participate because of their child’s illness and felt pressured to participate by their health care providers [18].

Only 9.4% of the study participants signed the consent form during the epidemiological survey. This is a far lower rate compared to other studies where most of the participants signed [19]. Unless waived by the ethics committee where research has no more than minimal risk, a consent form is required to be signed by the participant or guardian in the case of underage participants [17]. It is also noted that oral consent was considered more pragmatic while doing research in conflict areas involving semi-illiterate research participants [32]. This holds true in the current study, where the majority of our participants had no formal education or attended only primary school.

Due to the lack or unavailability of papers evaluating the quality of consent in conflict zones, all of the mentioned articles for comparison were trial studies, some of them with smaller sample sizes, and they took place in controlled clinical research settings. A study reported on summary of understanding capacity of consent by study participants yielded a range of 52.1–75.8% for major comprehension domains of consent which agrees figuratively with current study [33]. However, given our study is a community-centered observational epidemiological study that was conducted in conflict-affected areas, greatly differs from the nature and context of the cited research above. Therefore, this may challenge the head-to-head comparison on some of the figures, and the results should be taken cautiously for interpretation.

Despite the importance of this paper in addressing less studied areas of consent for research in conflict areas, we declare it is not free of limitations. This study had a shorter time interval of below two months compared to other studies aimed at assessing the quality of consent, which reported fourteen months and beyond in the interval between the real and consent studies. However, as long as the study was not a real-time assessment, it was still subject to recall bias. Hence, the information provision section shall be interpreted with care. Additionally, under the comprehension section, the response options were closed-ended, which is limited to measuring the comprehension capacity of the respondents. Therefore, we advise that the results be considered cautiously while interpreting and comparing.

## Conclusion

Despite its limitations, the current research has highlighted the level of adherence to ethical requirements in community-based epidemiological research, where conflict is common. The assessment in this study has clearly indicated that the information provided and the comprehension capacities of the consenting participants tend to be low. Therefore, consent validity in community-based studies conducted in conflict-prone areas is vulnerable to compromise. Although individual consenting is fundamental to considering ethical concerns, consent seeking in conflict settings need to be supplemented with other means of authorization such as community consultation in order to enhance the acceptance and comprehension of consenting. Given the identified low levels of information comprehension among consenting participants, it is crucial to enhance the informed consent process. Researchers should consider the use of clear, concise and diagram supported consent documents to aid in communication. Providing supplementary materials or offering opportunities for participants to ask questions and seek clarification can also enhance comprehension. The study may further necessitate the call to local and national Institutional Review Boards (IRB) to reframe ethical requirements to classify persons in conflict areas as vulnerable populations to ensure greater protection of their rights as research participants and a fair distribution of research benefits. It is recommended to promote the use and optimization of context-specific informed consent. Improved monitoring of research ethics integrity in resource-constrained and conflict-beset contexts should be enacted to solve observed ethical issues.

### Electronic supplementary material

Below is the link to the electronic supplementary material.

Supplementary Material 1

## Data Availability

The datasets used for the current study are available from the corresponding author upon reasonable request.

## Abbreviations

CI: Confidence Interval
IRB: Institutional Review Board
LMIC: Low- and Middle-Income Countries
MCAR: Missing Completely at Random
SD: Standard Deviation
