# Supplementary material for: Compliance with research ethics in epidemiological studies targeted to conflict-affected areas in Western Ethiopia: validity of informed consent (VIC) by information comprehension and voluntariness (ICV)
Source: BMC Med Ethics. 2024 Jan 18;25:9. doi: 10.1186/s12910-024-01003-5 (PMC10795316; doi:10.1186/s12910-024-01003-5)
Supplement: Supplementary file 1 — Supplementary Material 1 [file 12910_2024_1003_MOESM1_ESM.docx]

**Supplementary File 1: Survey Guide**

**Compliance with research ethics in Epidemiological Studies targeted to conflict affected areas at western Ethiopia: Validity of Informed Consent (VIC) by Information Comprehension and Voluntariness (ICV)**

| **Part A: Socio-demographic Information of the Research Participants** | |
| --- | --- |
| Variable | Codes/ Categories |
| Districts of data collection | Jima Arjo |
|  | Leka Dulecha |
|  | Wayu Tuka |
| Age in years |  |
| Sex of the respondents | Female |
|  | Male |
| Marital status | Married |
|  | Single |
| Educational attainment | No formal education attended |
|  | Primary school attended (1-8) |
|  | Secondary School attended (9-12) |
|  | College and above |
| Occupation | Daily laborer |
|  | Farmer |
|  | Government Employee |
|  | Housewife |
|  | Merchant |
| Religion | Orthodox |
|  | Protestant |
| **Part B: Type of Information Provided to the Participants enrolled in Epidemiological studies** | |
| Did the investigator tell why you were eligible for selection? | Yes |
|  | No |
| Did the investigator tell you the purpose of the research? | Yes |
|  | No |
| Did the investigator tell you procedures to be carried out? | Yes |
|  | No |
| Were told about autonomous participation and to withdraw if not interested? | Yes |
|  | No |
| Did the investigator tell you about the confidentiality of the information you provide? | Yes |
|  | No |
| Did the investigator tell you of any benefit of taking part in the research? | Yes |
|  | No |
| Were you informed of discomfort or pain, any risk of taking part in the research? | Yes |
|  | No |
| Did the investigator proposed to tell you of status of your health after research completion? | Yes |
|  | No |
| **Part C: Comprehension of the consented study participants** | |
| Differentiation of the research and routine care | Data collection for research |
|  | Part of health care service |
|  | Health related campaign |
| Was there any possibility of withdrawal after your enrollment? | Free to withdraw at any stage |
|  | Free to withdraw if granted permission only |
|  | No option of withdrawal once enrolled |
| Were you familiar with any anticipated risks as result of your participation in the research? | The procedure was non-invasive and painless |
|  | The procedure was painful |
|  | Did not understand any associated risks |
| Were you aware about the benefits of partaking in the research? | No direct benefit of partaking |
|  | The research findings will beneficial to the underlying community |
|  | Not much conversant about the benefits |
| Did you realize how the information you provide handled in secret? | The data is handled securely |
|  | Un-named data is used |
|  | Not clearly understood |
| **Part D: Assessment of voluntarism of the consented participants** | |
| Your willingness to participate in the research | Voluntary Participation |
|  | In forced Participation |
|  | Pressurized |
|  | Influenced participation |
| Have you clearly expressed your consent to participate? | Yes |
|  | No |
| How did you express your consent? | Orally agreed |
|  | Signed to participate |
| Have you consented to offer biological specimen? | Yes |
|  | No |
